# Screening for primary creatine deficiencies in French patients with unexplained neurological symptoms

**DOI:** 10.1186/1750-1172-7-96

**Published:** 2012-12-13

**Authors:** David Cheillan, Marie Joncquel-Chevalier Curt, Gilbert Briand, Gajja S Salomons, Karine Mention-Mulliez, Dries Dobbelaere, Jean-Marie Cuisset, Laurence Lion-François, Vincent Des Portes, Allel Chabli, Vassili Valayannopoulos, Jean-François Benoist, Jean-Marc Pinard, Gilles Simard, Olivier Douay, Kumaran Deiva, Alexandra Afenjar, Delphine Héron, François Rivier, Brigitte Chabrol, Fabienne Prieur, François Cartault, Gaëlle Pitelet, Alice Goldenberg, Soumeya Bekri, Marion Gerard, Richard Delorme, Marc Tardieu, Nicole Porchet, Christine Vianey-Saban, Joseph Vamecq

**Affiliations:** 1 Hospices Civils de Lyon, Service Maladies Héréditaires du Métabolisme et Dépistage Néonatal, Groupement Hospitalier Est, Bron, 69677, France; 2 Département de Biochimie et Biologie Moléculaire, Laboratoire d’Hormonologie, Métabolisme-Nutrition & Oncologie (HMNO)–Centre de Biologie et Pathologie (CBP) Pierre-Marie Degand, CHRU Lille, Lille, 59037, France; 3 Mass Spectrometry Application Laboratory, University of Lille 2, Lille, 59045, France; 4 Metabolic Unit, Department of Clinical Chemistry, VU University Medical Center Amsterdam, Amsterdam, The Netherlands; 5 Centre de Référence des Maladies Héréditaires du Métabolisme, Hôpital Jeanne de Flandres, CHRU Lille, Lille, 59037, France; 6 Service de Neurologie Infantile, Hôpital Roger Salengro, CHRU Lille, Lille, 59037, France; 7 Service de neurologie pédiatrique, CHU de Lyon-GH Est - Hôpital Femme Mère Enfant, Bron Cedex, 69677, France; 8 Laboratory of Biochemistry, Necker – Enfants Malades Hospital and Université Paris Descartes, Paris, 75015, France; 9 Centre de Référence des Maladies Héréditaires du Métabolisme, Hôpital Necker des Enfants Malades and Université Paris Descartes, 149 rue de Sèvres, Paris, 75015, France; 10 Département de Biochimie-Hormonologie, CHU Hôpital Robert Debré, Paris, 75019, France; 11 Unité de Neurologie Pédiatrique, Département de Pédiatrie, Hôpital Raymond Poincare, Paris-IdF-Ouest University, Paris, France; 12 Laboratoire de Biochimie et Biologie Moléculaire, CHU Angers, Angers, 49033, France; 13 Service de Neuropédiatrie - CHU de Bicêtre, Le Kremlin Bicêtre Cedex, 94275, France; 14 Service de Neuropédiatrie, Hôpital Armand Trousseau, Groupement hospitalier universitaire Est, Paris, 75012, France; 15 Unité Fonctionnelle de Génétique Médicale AP-HP, Département de Génétique et Cytogénétique, Centre de Référence «Déficiences intellectuelles de causes rares », CRicm, UMR-S975, Groupe Hospitalier Pitié-Salpêtrière, Paris, F-75013, France; 16 Neuropédiatrie, CHRU Montpellier, & Inserm U1046, Université Montpellier 1 & 2, Montpellier Cedex 5, 34295, France; 17 Service Neuropédiatrie, AP-HM Hôpital de la Timone, Marseille Cedex 5, 13385, France; 18 Service de Génétique, CHU de Saint-Étienne Hôpital Nord, Saint-Etienne Cédex 2, 42055, France; 19 Service de génétique Centre hospitalier Felix Guyon (Saint-Denis) Bellepierre, Saint-Denis cedex, 97405, France; 20 Service de Neuropédiatrie, Hôpital de l’Archet 2, Nice Cedex 3, 06202, France; 21 Service de Génétique Médicale, CHU Ch. Nicolle, Rouen Cedex, 76031, France; 22 Institut de Biologie Clinique, CHU Ch. Nicolle, Rouen Cedex, 76031, France; 23 Service de Génétique, CHU Clémenceau, Caen, 14033, France; 24 Service de Pédopsychiatrie CHU Hôpital Robert Debré, Paris, 75019, France; 25 Service de Neuropédiatrie - CHU de Bicêtre, Le Kremlin Bicêtre Cedex, 94275, France; 26 Inserm, Laboratoire Externe, Département du Prof. Nicole Porchet, HMNO, Centre de Biologie et Pathologie (CBP) Pierre-Marie Degand, CHRU Lille, Lille, 59037, France

## Abstract

A population of patients with unexplained neurological symptoms from six major French university hospitals was screened over a 28-month period for primary creatine disorder (PCD). Urine guanidinoacetate (GAA) and creatine:creatinine ratios were measured in a cohort of 6,353 subjects to identify PCD patients and compile their clinical, ^1^H-MRS, biochemical and molecular data. Six GAMT [*N*-guanidinoacetatemethyltransferase (EC 2.1.1.2)] and 10 X-linked creatine transporter (SLC6A8) but no AGAT (GATM) [L-arginine/glycine amidinotransferase (EC 2.1.4.1)] deficient patients were identified in this manner. Three additional affected sibs were further identified after familial inquiry (1 brother with GAMT deficiency and 2 brothers with SLC6A8 deficiency in two different families). The prevalence of PCD in this population was 0.25% (0.09% and 0.16% for GAMT and SLC6A8 deficiencies, respectively). Seven new PCD-causing mutations were discovered (2 nonsense [c.577C > T and c.289C > T] and 1 splicing [c.391 + 15G > T] mutations for the *GAMT* gene and, 2 missense [c.1208C > A and c.926C > A], 1 frameshift [c.930delG] and 1 splicing [c.1393-1G > A] mutations for the *SLC6A8* gene). No hot spot mutations were observed in these genes, as all the mutations were distributed throughout the entire gene sequences and were essentially patient/family specific. Approximately one fifth of the mutations of *SLC6A8*, but not *GAMT*, were attributed to neo-mutation, germinal or somatic mosaicism events. The only SLC6A8-deficient female patient in our series presented with the severe phenotype usually characterizing affected male patients, an observation in agreement with recent evidence that is in support of the fact that this X-linked disorder might be more frequent than expected in the female population with intellectual disability.

## Introduction

Creatine is a physiological compound that was first isolated by the French chemist Eugène Chevreul in the 1830s [1]. It is supplied to the body both via endogenous biosynthesis and the diet [2-4]. Its name (the Greek ‘*kreas*’ means meat) originates from its abundance in muscle in which its energetic role has previously been documented [5-8]. Creatine can be phosphorylated by creatine kinases, leading to the storage of a high-energy phosphate bond of ATP in the form of phosphocreatine [3]. Upon demand, and due to the reversible activity of creatine kinases which can transform ADP back into ATP, phosphocreatine can release the stored energy [3]. This creatine/phosphocreatine cycle represents a vital reserve of energy in tissues with high energetic needs such as muscles and the brain [3]. In recent years, the physiological role of brain creatine has been extended to include the modulatory control of neurotransmission which, in addition to guanidinoacetate (GAA) toxicity, has improved our understanding of brain function and its severe impairment in disorders affecting creatine biosynthesis and transport [9-12].

The first step of creatine biosynthesis occurs essentially but not exclusively in the kidney and involves the transfer of an amidino group from arginine to glycine by the enzyme L-arginine/glycine amidinotransferase (AGAT) (EC 2.1.4.1) to form GAA and L-ornithine. Subsequently, *N*-guanidinoacetatemethyltransferase (GAMT) (EC 2.1.1.2) catalyzes the transfer of a methyl group from *S*-adenosyl methionine (SAM) on GAA. This second step occurs notably in the liver and leads to the formation of creatine and *S*-adenosylhomocysteine (SAH) [3]. Once synthesized, creatine may be released into the bloodstream. It can be internalized by cells via a specific plasma membrane Na^+^/Cl^-^ dependent creatine transporter (SLC6A8) and stored as phosphocreatine [3]. Finally, about 1.7% of the creatine/phosphocreatine pool undergoes spontaneous degradation each day forming creatinine that is excreted in urine [3]. Urinary excretion of creatinine is a function of body muscle mass and in practice is a useful marker of kidney function. Little or no creatine is taken up by the brain from the systemic bloodstream because the blood brain barrier is practically impermeable to creatine. The brain must therefore secure most of its requirements in creatine by endogenous synthesis [10,11,13]. The crucial role of SLC6A8, which has been reviewed recently [14], provides ground for understanding how its deficiency results in a creatine collapse in brain.

Extracerebral and intracerebral routes for creatine supplies concur and their respective contributions differ in the immature (i.e. fetal and perinatal) and mature brain. In the immature brain, the extracerebral supply is favored due to a better efficacy of SLC6A8 in the blood–brain barrier (BBB). This means that extraction of creatine from the blood is predominant over intracerebral creatine biosynthesis which is physiologically limited by low GAMT activity in the immature brain [14]. SLC6A8 deficiency affects the immature brain creatine content through impairment of BBB uptake. In the mature brain, the contribution of BBB SLC6A8 becomes physiologically lower and intracerebral biosynthesis becomes the major pathway for the supply of brain creatine [14]. SLC6A8 deficiency therefore affects the mature brain creatine content through impairment of intracerebral creatine biosynthesis as a result of reduced SLC6A8-driven transfer of GAA from brain GAA to creatine-synthesizing cells. Differences existing between the extracellular/intracellular routes for the brain creatine supply explain why creatine supplementation is more efficient in two other creatine disorders, AGAT and GAMT deficiencies, when given at a presymptomatic stage of the disease when BBB uptake of creatine is physiologically optimal [14,15]. An illustrated account of creatine biosynthesis and transport is provided in the Additional file 1.

Primary creatine deficiency disorders (PCD) are a new class of inborn errors of metabolism to which belong AGAT (OMIM 602360) and GAMT (OMIM 601240) deficiencies, both inherited as an autosomal recessive trait, and SLC6A8 [CT1, CRTR] deficiency (OMIM 300036), an X-linked disorder [10-12,16-20]. The three disorders share a relative reduction of brain creatine and phosphocreatine levels [19], and accordingly, the clinical presentation is predominated by neurological signs [10-12,16-20]. However, these disorders still remain under-diagnosed whether considered together [16-20] or individually (AGAT [21-25], GAMT [26,27] or CRTR [28-33] deficiency).

In this paper we present the results of a large retrospective study of urinary screening for PCD on 6,353 patients in whom the etiology of the underlying neurological disease was unknown at the time of urinary analysis of creatine and its metabolites.

## Patients and methods

We retrospectively collected the metabolic results of urinary screening for PCD (GAA and creatine:creatinine ratios) from 6,353 patients with neurological disease of unknown origin (4,426 male patients and 1,927 female patients) hospitalized in six French university hospitals (Angers, Lille, Lyon and Paris (Hôpital Necker Enfants Malades, Hôpital Robert Debré and Hôpital Raymond Poincaré)) over a period of 28 months. Data with regard to GAA and creatine levels in plasma were also collected when available. For all patients with an abnormal result, a second sample was analyzed after a 24-hour diet devoid of meat and fish according to the recommendation of Arias *et al*. [34]. A patient was considered “at risk of PCD” (AGAT, GAMT or SLC6A8 deficiency) when the biochemical abnormality was confirmed on this second sample. Subsequently a molecular study and/or a functional test were performed to confirm the diagnosis. For the genetic study, informed consent was obtained from each patient or from their parents if probands were under 18. For patients with a confirmed diagnosis of PCD, extensive clinical data were collected, including familial, obstetrical and personal histories, main symptoms having led to consult, the specialty of the physician who requested metabolic investigations, the patient’s age at diagnosis, clinical signs present at diagnosis and results of ^1^H-MRS exploration. All patients with other diagnosed conditions (in particular, urea cycle disorders and remethylation defects) were discarded from the study.

### Methods for metabolite measurements

Creatine and GAA levels were measured in urine and plasma by tandem mass spectrometry (LC-MS/MS) using stable isotopes as internal standards (^13^C_2_-GAA and ^2^H_3_-creatine) according to a method described previously [35]. Urinary creatinine was measured using LC-MS/MS using a specific internal standard (^2^H_3_-creatinine), except in two university hospitals in Paris (Necker Enfants Malades and Robert Debré) where this measurement was performed using the colorimetric Jaffé method [36,37]. The control values and age groups used for plasma and urine GAA and creatine concentrations were those published by Verhoeven *et al*. [38].

### Measurement of protein activities

GAMT activity was determined in lymphoblasts by assaying the transfer of methyl groups from ^2^H_3_-S-adenosylmethionine to ^13^C_2_-labelled GAA through the production of ^13^C_2_^2^H_3_-creatine quantitated by an isotope dilution electrospray tandem spectrometry assay [39].

Creatine transporter activity was assayed by determining the intracellular incorporation of creatine in cultured skin fibroblasts as previously described [32].

### Genetic studies

Genomic DNA was isolated from white blood cells or cultured fibroblasts collected from patients after informed consent. The coding regions and adjacent intronic splice sites of the *GAMT* and *SLC6A8* genes were analyzed by direct sequencing. The pathogenicity of previously non-described mutations was predicted using the Alamut v2.02 software (Interactive Biosoftware, Rouen France) and compared to the LOVD database [40].

## Results

### Results of the screening for PCD in the French patients with unexplained neurological symptoms

Among the 6,353 patients screened, 16 patients were diagnosed to have PCD: 6 with GAMT deficiency (4 male and 2 female patients) and 10 with SLC6A8 deficiency (9 male and 1 female patients). Three additional affected sibs were identified after familial inquiries (1 brother affected with GAMT deficiency and 2 brothers with SLC6A8 deficiency in two different families). Diagnosis was confirmed by genetic analysis in 17 patients. For the other 2 patients (1 with GAMT deficiency and 1 with SLC6A8 deficiency), DNA analysis was rejected by the parents and the diagnosis was confirmed by the absence of a creatine peak in brain ^1^H-MRS, the urinary profile and the deficiency of protein activities. No patients with AGAT deficiency were identified in our cohort. The medical investigations leading to the diagnosis of these pathologies were requested by pediatric neurologists (42%), geneticists (32%), pediatricians (10.5%), adult neurologists (10.5%) and child psychiatrists (5%). Clinical descriptions and the results of the diagnostic explorations performed for the index patients and for the affected sibs are summarized in Table 1. The global prevalence of PCD in the screened population was 0.25% (16/6,353; CI95: 0.13% - 0.38%), the prevalence of GAMT deficiency was 0.09% (6/6,353; CI95: 0.02% - 0.17%) and the prevalence of SLC6A8 deficiency was 0.16% (12/6,353; CI95: 0.06% - 0.26%). Regarding our data, the prevalence of AGAT deficiency would be lower than 1/6,353, i.e. < 0.02% since no AGAT patient was diagnosed in the present study. The prevalence of PCD was 0.29% for the male patients (4 GAMT and 9 SLC6A8 deficiencies, 13/4,426; CI95: 0.14% - 0.46%) and 0.16% for the female patients (2 GAMT and 1 SLC6A8 deficiencies, 3/1,927; CI95: 0.0% - 0.34%). For the X-linked disorder SLC6A8 deficiency, the prevalence was 0.20% for the male patients (9/4,426; CI95: 0.08% - 0.34%) and 0.05% (1/1,927; CI95: 0.0% - 0.16%) for the female patients.

**Table 1:** **Main clinical, biological, genetic and**^**1**^**H**-**MRS features of the patients affected with PCD**

	**GAMT(n = 7)**	**CRTR(n = 12)**
**CLINICAL DATA**		
**Sex**		
Male	5/7	11/12
Female	2/7	1/12
*Ratio* (*M*/*F*)	*2*.*5*	*11*
**Consanguinity**	+++	-
**Age of onset** (years)		
≤ 2 years	6/7	12/12
> 2 years	1/7	0/12
**Age at diagnosis**(**years**)		
≤ 2 years	1/7	0/12
[2–5 years]	3/7	3/12
[5–10 years]	0/7	4/12
> 10 years	3/7	5/12
**Main clinical signs**		
Intellectual disability	++++	++++
Speech delay	++++	+++
Failure to thrive	+	+
Hypotonia	++	+
Myopathy	+	-
Motor delay	++	+++
Epilepsy	++	+
Extrapyramidal signs	+	-
Attention deficit	++++	+++
Sleeping disturbances	++	+
Agressive behaviour	+++	-
Autistic behaviour	++	+
**Signs leading to consult**		
Combined motor and speech delay	+++	+++
Epilepsy	++	+
Intellectual disability	+	-
Autistic behavior	-	++
Familial exploration	+	+
**METABOLIC DATA**		
**Plasma GAA** (μmol/l)		
<*15 years*: [*0*.*35*-*1*.*8*]^*a*^	[14–23]	[0.8 - 2.5]
>*15 years*: [*1*.*0*-*3*.*5*] ^*a*^	[20-23]	2.9
**Plasma creatine** (μmol/l)		
<*10 years*: [*17*–*109*]^*a*^	[3.3 - 10]	59; 65
>*10 years*: [*6*.*0*-*50*]^*a*^	[4–6]	100; 112
**Urine GAA**/**creatinine** (mmol/mol)		
<*15 years*: [*2*–*220*]^*a*^	[399–1319]	[30–219]
>*15 years*: [*3*–*78*]^*a*^	242; 558	37; 38
**Urine creatine**/**creatinine** (mmol/mol)		
<*4 years*: [*6*–*1208*]^*a*^	11; 33	2762
*4*–*12 years*: [*17*–*721*]^*a*^	18; 26	[1638–3015]
>*12 years*: [*11*–*244*]^*a*^	[12–32]	[1181–3195]
**Brain**^**1**^**H**-**MRS investigation**	Absence of creatine peak (n = 5)	Absence of creatine peak (n = 6)
**GENETIC DATA**		
Non-sense mutation	2/6	0/11
Missense mutation	1/6	2/11
Insertion	0/6	0/11
Deletion	0/6	6/11
Duplication	2/6	0/11
Splicing mutation	1/6	3/11
**FUNCTIONNAL TESTS**		
GAMT Activity (lymphoblasts)	Deficiency (2/2)	/
Creatine transport assay (fibroblasts)	/	Deficit of transport (3/3)

This table groups data from the diagnosed PCD patients and affected siblings. Fraction numbers refer to the number of positive patients (numerator) on the total number of patients studied (denominator) for the indicated item. Frequencies of signs in patients are expressed as + and – symbols: ++++, presence of signs in all patients; -, absence of signs in all patients; +++, ++ and +, presence of the sign in more than 75%, between 25 to 75%, and less than 25% of patients, respectively. ^a^ Reference laboratory values and age ranges are those published by Verhoeven *et al*. [38], patient data being expressed as either range or individual values. GAMT, guanidinoacetate methyltransferase; CRTR, creatine transporter SLC6A8.

### Patients with GAMT deficiency

Seven patients from 6 unrelated families were diagnosed with GAMT deficiency. The sex ratio (M/F) was 2.5. Five of the 7 patients were born of a consanguineous union. Most of the patients were of Caucasian origin. Obstetrical history was available for 5 of the 7 patients; pregnancies were normal without pre-term birth. The mean ± SD values for birth-related parameters were: gestational term, 39.1 ± 0.5 weeks; birth weight, 3192 ± 135 g; birth length, 49.4 ± 1.4 cm and head circumference, 34.0 ± 1.2 cm. The predominant clinical signs at diagnosis were intellectual disability (100%) and speech delay (100%) (for general information on speech development and delay at a young age, see [41,42]), and were associated with epilepsy (57%), motor delay (43%), hypotonia (43%) and extrapyramidal symptoms (29%). Behavioral disturbances were noticed in all patients essentially under the form of attention deficit (100%) with aggressive behaviors (hetero- and/or auto-mutilating episodes, anger and intolerance to frustration) (86%), autistic behavior (43%) and sleeping disturbances (43%). Most of these signs were usually present before 2 years of age and signs leading to consult are detailed in Table 1.

Diagnosis of GAMT deficiency was established at a median patient age of 5 years, with age values ranging between 2 and 29 years. In urine, the GAA:creatinine ratio (U-GAA/CTN) was increased for all patients and exceeded the upper control limits by 1.8- to 7.2-fold whereas the creatine:creatinine ratio (U-CT/CTN) was normal. When assayed, plasma GAA levels were systematically increased and creatine levels were below or equal to the lowest values of control limits. Cerebral ^1^H-MRS was performed in 5 patients and showed that creatine was undetectable in the brain in all the cases. In 2 of the 7 patients, GAMT activity was measured in lymphoblasts and these enzyme studies validated GAMT deficiency. The *GAMT* gene was studied in 6 of the 7 patients. In each case, patients had presumed (parents not available for study) or confirmed (parents were heterozygous for the mutation) homozygous mutations (Table 2). Mutations were either missense (n = 1), nonsense (n = 2), frame shift (n = 2) or splicing (n = 1) mutations.

**Table 2:** **Mutations in the *GAMT *gene**

**Mutations**			**Number of patients identified in this study**		**Reference**
**Exon (e)/Intron (i)**	**Nucleotide**	**Amino acid**	**Homozygous**	**Heterozygous**	
e2	c.289C > T	p.Q97X	1	-	This study
e2	c.299_311dup13	p.R105GfsX26	2^a^	-	Dhar *et al*., 2009 [27]
i3	c.391 + 15G > T	p.(?)	1	-	This study
e5	c.506G > A	p.C169Y	1	-	Caldeira Araujo *et al*., 2005 [63]
e6	c.577C > T	p.Q193X	1	-	This study

Nucleotide numbering starting at the first adenine of the translation initiation codon ATG.

^a^ Two affected siblings.

### Patients with SLC6A8 deficiency

Twelve patients from 10 unrelated families were identified with SLC6A8 deficiency. The sex ratio (M/F) was 11. When available, the notion of consanguinity was absent. Obstetrical history was available for one patient with a gestational term at 39 weeks; birth weight, 3080 g; length, 48 cm, head circumference, 32 cm. At diagnosis, intellectual disability (100%) associated with language (75%) or motor (83%) delays were reported in most of the patients. Behavioral abnormalities were observed in 83% (10/12) patients and evoked those seen in GAMT deficiency patients, notably attention deficit (83%, 10/12 patients) with hyperactivity, autistic behavior (17%) and sleeping disturbances (8%). No aggressive behavior was observed. Epilepsy was reported in 17% of the patients. The main signs leading parents to consult (see Table 1 for an overview) were combined motor and speech delays; autistic behavior was the second cause of consultation. Familial inquiries led to the diagnosis of SLC6A8 deficiency in 2 of the 12 patients having an affected sibling. Anamnesis indicated that the initial signs could be present before the age of 2 years with in particular global psychomotor retardation and to a lesser extent epilepsy and autistic behavior. The median age for diagnosis was 9.5 years with age values ranging between 2.5 and 28 years.

The U-CT/CTN ratio was increased in all male patients, and exceeded the upper control limit by 2.3- to 13.6-fold. For the affected girl, the U-CT/CTN ratio was only 2.3-fold greater than the upper control limit. In plasma, creatine levels were normal or moderately increased (up to 1.3-fold greater than the upper control limit value). Plasma GAA levels were normal (or slightly affected), as were urinary U-GAA/CTN ratios. Cerebral ^1^H-MRS was performed in 50% (6/12) of the patients and consistently confirmed creatine deficiency in the brain in all examined patients (undetectable creatine peak). In 3 of the 12 (25%) patients, a functional study of SCL6A8 activity was carried out in cultured skin fibroblasts, and evidenced a deficiency of the creatine transporter function.

The *SCL6A8* gene was studied in 92% (11/12) of the patients (Table 3). A hemizygous mutation was identified for all the affected male patients. For the female patient, a pathogenic mutation was identified at a heterozygous state and no X-inactivation biases could be identified in leucocytes. Mutations were either missense (n = 2), frame shift (n = 6) or splicing (n = 3) mutations. The underlying mutations were also detected in the mothers of the patients, except for the heterozygous female patient (c.942_944delCTT in exon 6) and a hemizygous boy (c.1208C > A in exon 8). In the latter two patients, SLC6A8 deficiencies were concluded to result from neo-mutation, germinal or somatic mosaicism.

**Table 3:** **Mutations in the *SLC6A8 *gene**

**Mutations**			**Number of patients identified in this study**		**Reference**
**Exon (e)/Intron (i)**	**Nucleotide**	**Amino acid**	**Male**	**Female**	
			**Hemizigous**	**Heterozygous**	
e2	c.321_323delCTT	p.F107del	2 ^a^	-	Degrauw *et al*., 2002[64]
i4	c.778-2A > G	p.(?)	1	-	Betsalel *et al*., 2011[61]
e6	c.926C > A	p.A309E	1	-	This study
e6	c.930delG	p.T311PfsX85	1	-	This study
e6	c.942_944delCTT	p.F314del	-	1	Fons *et al*., 2009[65]
e6	c.1006_1008delAAC	p.N336del	1	-	Clark *et al*., 2006[56]
e8	c.1208C > A	p.A403D	1	-	This study
i9	c.1393-1G > A	p.(?)	2 ^a^	-	This study
e11	c.1519_1543del	p.I507LfsX5	1	-	Betsalel *et al*., 2011[61]

Nucleotide numbering starting at the first adenine of the translation initiation codon ATG.

^a^ Two affected brothers.

## Discussion

In the present study we have identified 16 new patients diagnosed with a PCD over a period of 28 months, as well as three affected siblings. Intellectual disability and attention deficit were, along with motor and speech delays, the main symptoms reported by the medical staff involved in the diagnosis of these disorders. Nevertheless, it is surprising that intellectual disability, which was a sign observed by the medical examiners in all patients with PCD, was not the main cause leading the parents to consult. This might reflect the difficulty of identifying intellectual disability in the very young child or in turn to of associating intellectual disability with PCD. These disorders are known to progress via irreversible lesions of the brain, so therapeutic measures are needed to be taken early in the life of the patients. For GAMT patients, supplementation with creatine together with a GAA lowering strategy (ornithine supplementation with or without arginine restriction) may represent an efficient treatment strategy [43-46] and may provide some medical neurological benefit for GAMT patients. In these conditions, patients may recover a normal brain creatine peak as detected by ^1^H-MRS as well as normal urine GAA levels. They also improve clinically with better language and social development and apparently some regression of the epilepsy [47]. By contrast, in patients with SLC6A8 deficiency, supplementations with creatine and its precursors arginine or glycine, though improving muscular signs, were found to be without substantial benefit on cognitive and psychiatric signs, and failed to modify the creatine signal in ^1^H-MRS imaging of the brain [48], suggesting the inefficacy of these supplementations in improving brain as stated by other studies [4,19,30,49-52]. Though there is hope to treat efficiently this group of patients through the discovery of creatine pro-drugs capable of entering the brain and cells via a by-pass of the SCL6A8 creatine transporter, some clinical studies also question the inefficacy of the therapeutic measures mentioned above and, on the contrary, show beneficial effects with creatine and/or arginine supplementations in patients with SLC6A8 deficiency. In a heterozygous female patient with intractable epilepsy, treatment with creatine combined with arginine and glycine completely resolved seizures [53]. Creatine supplementation was also described to improve the neurological, language and behavioral status and was associated with a rise in the brain creatine peak as demonstrated by MRS in a child with SLC6A8 deficiency [54]. In a recent study, creatine deficient patients were also shown to be improved by a L-arginine-based therapy which positively impacted daily living skills, lowered the frequency of epileptic episodes and induced a mild increase in brain creatine and phosphocreatine MRS signals although normal cerebral levels of these metabolites were not recovered [55].

The calculated prevalence of PCD in our cohort was 0.25%. This result was not expected because most studies report a higher prevalence of PCD, notably SLC6A8 deficiency, which is generally estimated between 1% and 3% of the population affected with intellectual disability [56-59]. However, this prevalence has been considered to be an overestimation of the real prevalence of PCD. Because PCDs are monogenic disorders, their prevalence was proposed to be no different from that of other nonsyndromic diseases such ARX (Aristaless-Related homeobox gene located on X chromosome) and therefore closer to 0.1% - 0.3% [60]. Thus, for the first time, our screening study provides strong practice-based evidence confirming this estimated prevalence for PCD. The girl diagnosed with SLC6A8 deficiency presented a severe phenotype similar to affected male patients, an observation that is not surprising in view of recent work [34,61,62] also describing this X-linked disorder in the female population with intellectual disability. In this respect, screening for SLC6A8 deficiency in female patients should be included in the diagnostic workup since this disorder still remains under-diagnosed. However, it should be noted that if the urinary U-CT/CTN ratio is not increased the diagnosis cannot be ruled out because it has been shown that the majority of female patients with a heterozygous mutation in SLC6A8 have a normal ratio, although the average ratio of this group is increased [62]. It might be recommended to perform brain MRS and/or SLC6A8 gene studies in female patients with suspected creatine transporter deficiency.

Five mutations were identified for *GAMT* gene in 6 unrelated families. These included 3 new mutations, c.289C > T, c.391 + 15G > T and c.577C > T, and two previously described mutations, c.299_311dup13 [27] and c.506G > A [63] (Table 2). The new mutation c391 + 15G > T was considered pathogenic by the creation of an alternative splicing donor site in intron 3 (Alamut software), by the segregation of this mutation in his parents and because it was linked with decreased GAMT activity in cultured fibroblasts.

The *SLC6A8* gene was analyzed in 11 patients from 9 unrelated families. Nine mutations were identified throughout the gene, including 4 new mutations (2 missense (c.1208C > A and c.926C > A), 1 frameshift (c.930delG) and 1 splicing (c.1393-1G > A) mutation), and 5 previously described mutations (c.321_323delCTT [64], c.942_944delCTT [65], c.1006_1008delAAC [56], c.778-2A > G and c.1519_1543del [61]) (Table 3). In accordance with the results of Clark *et al*. [56], we found a fairly high proportion of frameshift and splicing mutations in our patients, and approximately one fifth of the mutations of *SLC6A8*, but not *GAMT*, were attributed to neo-mutation, germinal or somatic mosaicism events.

Interestingly, DNA studies performed in our patients clearly showed that mutations were essentially patient/family specific and highlighted the absence of hot spot mutations in these genes. Finally, as a mutation was identified in each patient for whom DNA was analyzed, the study of the target gene appears to be a key diagnostic step for PCD in suspect patients, keeping in mind that secondary creatine disorders such as deficiencies of ornithine delta-aminotransferase (EC 2.6.1.13) [66,67], urea [68-71] and homocysteine/methionine [72,73] cycles, and succinate semialdehyde dehydrogenase (SSADH) (EC 1.2.1.24) [74,75] may also be alternative causes of abnormal laboratory values of creatine metabolism markers.

## Conclusion

Screening for PCD in the population with neurological disorders of unknown etiology has shown that these patients express important clinical signs of PCD which mainly include intellectual disability, speech delay, epilepsy, attention deficit and autistic behavior.

In this respect, some improvement might be made by conducting an information campaign among general and specialized educational staff to contribute to better recognizing intellectual disability and hence earlier referral of the child for professional medical evaluation. Improved anamnesis in regard to the familial history of possible patients might be also helpful. As suggested in the present study and highlighted in other work, both female and male patients can be concerned by severe PCD. However, and importantly, our study has shown that the prevalence of PCD remains low in the patients with unexplained neurological symptoms. Finally, the mutational spectrum of PCD has been extended by the new mutations detected during the present screening effort.

## Consent

When PCD was suspected informed consent was obtained from patients and/or their parents for the DNA analysis of *GAMT* or *SLC6A8* genes.

## Competing interests

The authors declare that they have no competing interests.

## Authors’ contributions

DC and MJCC contributed equally to this work and are first co-authors. Authors (DC, MJCC, GB, KMM, DD, JMC; AC, VV; JFB; JMP, GSI, OD, AA, LL, VD, MG, RD, NP, JV) working in the six major French university hospitals (including those of Lille, Lyon and Angers and the Hôpital Necker Enfants Malades, Hôpital Robert Debré and Hôpital Raymond Poincaré from the Public Assistance of Parisian Hospitals) collaborated to group and to generate clinical biological, biochemical and genetic data. The other authors were also involved in the clinical follow-up (AG, GP, BC, FR, KD, MT) or biological chemistry (SB) of some of the patients and in the gene studies (FC, FP, DH and GSA). DC, MJCC, GB, NP and JV analyzed and integrated the clinical and paraclinical data. JV coordinated the writing of the manuscript with DC, MJCC and GB. All authors read and approved the final manuscript.

## Supplementary Material

Additional file 1 **Updated view of creatine metabolism and transport.** Two enzymes are involved in the biosynthesis of creatine: arginine-glycineamidinotransferase (AGAT) and guanidinoacetatemethyltransferase (GAMT). A third protein, a plasma membrane transporter (SLC6A8), supplies the cell with creatine. A deficiency of any of these three biosynthesis and transport steps (namely, AGAT deficiency, GAMT deficiency and creatine transporter deficiency) leads to a deficiency of creatine in the brain (collapse of brain content in creatine). Biosynthesis of creatine happens in organs that contain both AGAT and GAMT. However, particularly in the brain, AGAT and GAMT may be distributed in different cells, so the guanidinoacetate (GAA) formed in one cell can be further transformed into creatine in another cell. This requires, as illustrated in the figure, the creatine transporter which can supply cells not only with creatine but also with GAA. The reason for collapse in brain creatine in either AGAT or GAMT deficiency is easily understood by a severe impairment of one of the two creatine biosynthesis steps. In creatine transporter deficiency, several facts concur including : (*i*) heterogeneity of brain cells for their content in AGAT and GAMT, (*ii*) most cerebral cells contain only of one of these two creatine biosynthesis enzymes; (*iii*) the blood–brain barrier is weakly permeable to systemic creatine and the brain must secure most of its creatine requirements by endogenous synthesis; and (*iv*) the creatine transporter contributes in the brain not only to supplying the cells with creatine but also directly to creatine biosynthesis by bridging the formation of an intermediate (GAA) in one cerebral cell to its utilization (towards creatine synthesis) in another cerebral cell. Another protein, the taurine transporter has also been shown to be active on GAA but in this case it leads to its removal from brain structures and CSF into blood, a feature having GAA detoxifying properties, notably in GAMT deficiency. Also depicted in the figure is the creatine kinase (CK) system (based on phosphocreatine as a stored form of energy produced from and capable of restoring ATP) and the non-enzymatic formation from creatine and phosphocreatine of creatinine (an end product of creatine metabolism removed in body fluids). Not depicted in the figure is the transit via body fluids which may take place in intercellular transfer and removal of creatine metabolites. For additional considerations and references, the reader is kindly referred to the recent review of Olivier Braissant (Ref [14] in the text). Click here for file
