# Supplementary material for: Screening for primary creatine deficiencies in French patients with unexplained neurological symptoms
Source: Orphanet J Rare Dis. 2012 Dec 13;7:96. doi: 10.1186/1750-1172-7-96 (PMC3552865; doi:10.1186/1750-1172-7-96)
Supplement: Additional file 1 — Updated view of creatine metabolism and transport. Two enzymes are involved in the biosynthesis of creatine: arginine-glycineamidinotransferase (AGAT) and guanidinoacetatemethyltransferase (GAMT). A third protein, a plasma membrane transporter (SLC6A8), supplies the cell with creatine. A deficiency of any of these three biosynthesis and transport steps (namely, AGAT deficiency, GAMT deficiency and creatine transporter deficiency) leads to a deficiency of creatine in the brain (collapse of brain content in creatine). Biosynthesis of creatine happens in organs that contain both AGAT and GAMT. However, particularly in the brain, AGAT and GAMT may be distributed in different cells, so the guanidinoacetate (GAA) formed in one cell can be further transformed into creatine in another cell. This requires, as illustrated in the figure, the creatine transporter which can supply cells not only with creatine but also with GAA. The reason for collapse in brain creatine in either AGAT or GAMT deficiency is easily understood by a severe impairment of one of the two creatine biosynthesis steps. In creatine transporter deficiency, several facts concur including : (i) heterogeneity of brain cells for their content in AGAT and GAMT, (ii) most cerebral cells contain only of one of these two creatine biosynthesis enzymes; (iii) the blood–brain barrier is weakly permeable to systemic creatine and the brain must secure most of its creatine requirements by endogenous synthesis; and (iv) the creatine transporter contributes in the brain not only to supplying the cells with creatine but also directly to creatine biosynthesis by bridging the formation of an intermediate (GAA) in one cerebral cell to its utilization (towards creatine synthesis) in another cerebral cell. Another protein, the taurine transporter has also been shown to be active on GAA but in this case it leads to its removal from brain structures and CSF into blood, a feature having GAA detoxifying properties, notably in GAMT de [file 1750-1172-7-96-S1.doc]

Additional file 1

**Additional file 1 – Updated view of creatine metabolism and transport**

Two enzymes are involved in the biosynthesis of creatine: arginine-glycineamidinotransferase (AGAT) and guanidinoacetatemethyltransferase (GAMT). A third protein, a plasma membrane transporter (SLC6A8), supplies the cell with creatine. A deficiency of any of these three biosynthesis and transport steps (namely, AGAT deficiency, GAMT deficiency and creatine transporter deficiency) leads to a deficiency of creatine in the brain (collapse of brain content in creatine). Biosynthesis of creatine happens in organs that contain both AGAT and GAMT. However, particularly in the brain, AGAT and GAMT may be distributed in different cells, so the guanidinoacetate (GAA) formed in one cell can be further transformed into creatine in another cell. This requires, as illustrated in the figure, the creatine transporter which can supply cells not only with creatine but also with GAA. The reason for collapse in brain creatine in either AGAT or GAMT deficiency is easily understood by a severe impairment of one of the two creatine biosynthesis steps. In creatine transporter deficiency, several facts concur including : ***(i)*** heterogeneity of brain cells for their content in AGAT and GAMT, ***(ii)*** most cerebral cells contain only of one of these two creatine biosynthesis enzymes; ***(iii)*** the blood-brain barrier is weakly permeable to systemic creatine and the brain must secure most of its creatine requirements by endogenous synthesis; and ***(iv)*** the creatine transporter contributes in the brain not only to supplying the cells with creatine but also directly to creatine biosynthesis by bridging the formation of an intermediate (GAA) in one cerebral cell to its utilization (towards creatine synthesis) in another cerebral cell. Another protein, the taurine transporter has also been shown to be active on GAA but in this case it leads to its removal from brain structures and CSF into blood, a feature having GAA detoxifying properties, notably in GAMT deficiency. Also depicted in the figure is the creatine kinase (CK) system (based on phosphocreatine as a stored form of energy produced from and capable of restoring ATP) and the non-enzymatic formation from creatine and phosphocreatine of creatinine (an end product of creatine metabolism removed in body fluids). Not depicted in the figure is the transit *via* body fluids which may take place in intercellular transfer and removal of creatine metabolites. For additional considerations and references, the reader is kindly referred to the recent review of Olivier Braissant (Ref [14] in the text).
